# LINK-A: unveiling its functional role and clinical significance in human tumors

**DOI:** 10.3389/fcell.2024.1354726

**Published:** 2024-04-05

**Authors:** Bing Liao, Jialing Wang, Yilin Xie, Hongliang Luo, Jun Min

**Affiliations:** ^1^ Department of Otorhinolaryngology, The Second Affiliated Hospital, Jiangxi Medical College, Nanchang University, Nanchang, Jiangxi, China; ^2^ Department of Gastrointestinal Surgery, The Second Affiliated Hospital, Jiangxi Medical College, Nanchang University, Nanchang, Jiangxi, China; ^3^ Second School of Clinical Medicine, Jiangxi Medical College, Nanchang University, Nanchang, Jiangxi, China; ^4^ Department of Neurology, The Second Affiliated Hospital, Jiangxi Medical College, Nanchang University, Nanchang, Jiangxi, China

**Keywords:** lncRNA, LINK-A, biological roles, clinical applications, oncological marker

## Abstract

LINK-A, also recognized as LINC01139, has emerged as a key oncological lncRNA in cancer. LINK-A is upregulated in solid and liquid tumor samples, including breast cancer, ovarian cancer, glioma, non-small-cell lung cancer, and mantle cell lymphoma. Notably, LINK-A is involved in regulating critical cancer-related pathways, such as AKT and HIF1α signaling, and is implicated in a range of oncogenic activities, including cell proliferation, apoptosis, epithelial-mesenchymal transition (EMT), cell invasion and migration, and glycolysis reprogramming. LINK-A’s differential expression and its correlation with clinical features enable it to be a promising biomarker for cancer diagnosis, prognosis, and the stratification of tumor progression. Additionally, LINK-A’s contribution to the development of resistance to cancer therapies, including AKT inhibitors and immunotherapy, underscores its potential as a therapeutic target. This review provides a comprehensive overview of the available data on LINK-A, focusing on its molecular regulatory pathways and clinical significance. By exploring the multifaceted nature of LINK-A in cancer, the review aims to offer a valuable resource for future research directions, potentially guiding the development of novel therapeutic strategies targeting this lncRNA in cancer treatment.

## 1 Introduction

Recent advances in RNA sequencing (RNA-seq) technology have led researchers to an intriguing finding: about 98% of the human genome, though transcribed, does not encode proteins (Wilusz et al., 2009; Ozsolak and Milos, 2011; Dhanoa et al., 2018). Instead, these sequences are known as non-coding RNAs (ncRNAs). Among these, a specific group exceeding ∼200 nucleotides (nt) in length is identified as long non-coding RNAs (lncRNAs) (Chowdhary et al., 2021; Mattick et al., 2023). Extensive research has illuminated the significant roles of lncRNAs in gene regulation, spanning processes such as transcription, translation, and post-transcriptional modifications (Hung and Chang, 2010; He et al., 2019; Statello et al., 2021a; Karakas and Ozpolat, 2021). Moreover, lncRNAs have emerged as key players in various cellular activities (Zhang et al., 2013; Li et al., 2016; Lin et al., 2020; Wu et al., 2020; Liu et al., 2021), including tumor proliferation, growth, metastasis, metabolic regulation, immune modulation, and drug resistance. Their impact is significantly determined by their sub-cellular localization, which influences their interactions within the cell. Nuclear lncRNAs can alter gene expression (Sun et al., 2018), while cytoplasmic lncRNAs may affect mRNA stability and translation (Statello et al., 2021b; Herman et al., 2022). Recognizing their diverse roles, lncRNAs have also gained recognition as a new approach to gene therapeutic targets and biomarkers in cancer diseases (Arun et al., 2018; Qian et al., 2020; Nandwani et al., 2021; Badowski et al., 2022; Zhou et al., 2022; Tang et al., 2024).

The Long Intergenic Non-Coding RNA For Kinase Activation (LINK-A), also known as Long Intergenic Non-Protein Coding RNA 1139 (LINC01139), is located on chromosome 1q43. It comprises two exons and spans 5634 nucleotides. This approximately 1.5 kb lncRNA, LINK-A, was initially identified in 2006 (Bubb et al., 2006). Furthermore, computational analyses and characterizations of LINK-A have revealed that its transcript structure includes a stem-loop configuration (Lin et al., 2016; Lin et al., 2017; Filippov-Levy et al., 2020). This structure accommodates several protein-binding motifs, notably two for BRK, one each for LRRK2, PC, and PIP3(25-27), showcasing its complex regulatory potential in cellular signaling pathways.

LINK-A has been observed to exhibit abnormal regulation in tumor samples (Lin et al., 2017; Ma and Xue, 2018; Zhao et al., 2018; Zhang et al., 2019a; Hua et al., 2019), and LINK-A level in serum could serve as a diagnostic biomarker for cancer (Zhang et al., 2019a; Zhang et al., 2019b; Hua et al., 2019; Liu et al., 2019; Kong et al., 2020; Zhang et al., 2020). LINK-A plays a multifunctional role in the pathogenesis of diverse malignancies, including breast cancer (BC) (Lin et al., 2016; Lin et al., 2017; Hu et al., 2019), glioma (Wu et al., 2017; Hua et al., 2019), ovarian carcinoma (OC) (Filippov-Levy et al., 2018; Ma and Xue, 2018; Zhang et al., 2019b; Chen et al., 2019; Filippov-Levy et al., 2020; Maleki et al., 2020), non-small-cell lung cancer (NSCLC) (Zhao et al., 2018; Liu et al., 2019; Maleki et al., 2021), mantle cell lymphoma (MCL) (Zhang et al., 2019a; Zhang et al., 2021), osteosarcoma (Zhao et al., 2019; Kong et al., 2020), hepatocellular carcinoma (HCC) (Li et al., 2020; Tang et al., 2022), pancreatic adenocarcinoma (PDAC) (Zhang et al., 2020; Ghahfarokhi et al., 2023), and esophageal squamous cell carcinoma (ESCC) (Nan et al., 2023). The transition from normal to tumor phenotypes and across different tumor stages has been characterized in several studies (Zhao et al., 2018; Zhao et al., 2019; Nan et al., 2023). For instance, LINK-A levels were found to gradually increase from normal tissue to para-tumor and ESCC tissue (Nan et al., 2023), suggesting its involvement in disease progression. Similarly, plasma levels of LINK-A are elevated in osteosarcoma patients, with a significant progression from healthy individuals to those with non-metastatic and then metastatic osteosarcoma, linking it to tumor metastasis (Zhao et al., 2019). Additionally, LINK-A is overexpressed in NSCLC, and its levels were significantly correlated with larger tumor size, and higher tumor grade (Zhao et al., 2018). Moreover, LINK-A has emerged as a valuable biomarker for diagnosis and prognosis in multiple tumor types (Lin et al., 2016; Zhao et al., 2018; Zhang et al., 2019a; Zhang et al., 2019b; Hua et al., 2019; Liu et al., 2019; Zhao et al., 2019; Li et al., 2020; Zhang et al., 2020; Nan et al., 2023). This review represents the first comprehensive summary of the research progress, molecular regulatory mechanisms, and clinical significance of LINK-A. It is designed to highlight LINK-A’s critical role in cancer biology and offer insightful guidance for future research on this significant lncRNA.

## 2 LINK-A and its role in tumorigenesis and development

Extensive research has demonstrated that the dysregulation of LINK-A plays a crucial role in driving tumor progression (Lin et al., 2016; Lin et al., 2017; Wu et al., 2017; Ma and Xue, 2018; Zhao et al., 2018; Zhang et al., 2019a; Zhang et al., 2019b; Hu et al., 2019; Hua et al., 2019; Liu et al., 2019; Zhao et al., 2019; Filippov-Levy et al., 2020; Kong et al., 2020; Li et al., 2020; Zhang et al., 2020; Maleki et al., 2021; Zhang et al., 2021; Nan et al., 2023) (Table 1). In the majority of tumors studied, LINK-A acts as an oncogenic lncRNA, amplifying several cellular processes integral to cancer development (Table 1). These processes include proliferation, invasion, migration, epithelial-mesenchymal transition (EMT), tumor growth, and metastasis. It is also implicated in actively suppressing apoptosis, thereby aiding in the survival of cancer cells. Furthermore, LINK-A affects tumor growth by influencing glycolysis reprogramming, an essential component of cancer cells’ metabolic adaptation.

**TABLE 1:** Roles and mechanisms of dysregulation of LINK-A in different cancers.

Tumor types	Models	Cell lines	Animals	Regulatory mechanisms	Biological effects	References
Breast cancer	*In vitro* and *in vivo*	MDA-MB-231, SUM149, MDA-MB-157, MDA-MB-468, HCC-1806, MCF-7, BT474, SK-BR-3, MCF-10A	Female nude mice (6-8 weeks old)	HIF1α signaling	Glycolysis reprogramming, tumor growth	Lin et al. (2016)
	*In vitro* and *in vivo*	MDA-MB-231, MDA-MB-468, MCF-10A, DLD-1 PIK3CA^+/+^ and PIK3CA^+/−^ cells	Female athymic Nu/Nu mice (4-6 weeks old)	PIP3-AKT pathway	Drug resistance (AKT inhibitors), tumor growth	Lin et al. (2017)
	*In vitro* and *in vivo*	MDA-MB-231, MDA-MB-468, BT549, HCC-1187; BT474, SK-BR-3; MCF7, T47D, ZR-75–1; MCF10A, NMuMG; SUM-149, B16-OVA, and B16F10	Transgenic mouse models: MMTV-cre, MMTV-Tg(LINK-A) and Tg(LINK-A) mice	GPCR-PKA-TRIM71 signaling, cAMP/PKA pathway	Immuno-suppression, immunotherapy resistance, tumor growth and metastasis	Hu et al. (2019)
Glioma	*In vitro*	U87MG, U-251MG, Normal human astrocytes	–	LINK-A/LDH-A axis	Cell proliferation, migration, invasion, glycolysis	Wu et al. (2017)
	*In vitro*	CCD-25Lu, Hs 683	–	LINK-A/Survivin axis	Cell apoptosis	Hua et al. (2019)
Ovarian carcinoma	*In vitro*	OVCAR-3, OVCAR-8, OVCA433, OC-238, OVCA 429, DOV13 and ES-2	–	ERK1/2 phosphorylation	Spheroid formation, migration, invasion, matrix metalloproteinase activity	Filippov-Levy et al. (2020)
	*In vitro*	UWB1.289	–	HIF-1α pathway	Cell migration, invasion	Zhang et al. (2019b)
	*In vitro*	UWB1.289, UWB1.289+BRCA1, SV40	–	TGF-β pathway	Cell migration, invasion	Ma and Xue (2018)
Non-small-cell lung cancer	*In vitro* and *in vivo*	A549, H1703, SK-MES-1, NCI-H1299	Nude mouse xenograft model	LINK-A/Hexokinase-II axis	Cell proliferation, aerobic glycolysis, tumor growth	Zhao et al. (2018)
	*In vitro*	HBEC3-KT, H1993, H1581	–	Akt signaling	Cell migration, invasion	Liu et al. (2019)
	*In vitro*	A549, Calu-3	–	HIF1α signaling	Cell viability, proliferation, cell migration, invasion, apoptosis, cell cycle progression	Maleki et al. (2021)
Mantle cell lymphoma	*In vitro*	JVM-2, Z-138	–	LINK-A/Survivin axis	Cell proliferation, apoptosis	Zhang et al. (2019a)
	*In vitro*	MAVER-1, Granta-519, Mino, REC-1, Jeko-1	–	AKT/Bcl2 pathway	Cell viability, cell apoptosis, chemoresistance (Ibrutinib)	Zhang et al. (2021)
Osteosarcoma		MG-63, U2OS	–	LINK-A/HIF1α axis	Cell migration, invasion	Zhao et al. (2019)
	*In vitro*	MG-63, U2OS	–	LINK-A/TGF-β1 axis	Cell migration, invasion, stemness	Kong et al. (2020)
Hepatocellular carcinoma	*In vitro*	LO2, Hep3B, QSG 7701, MHCC97H, Huh7, HepG2, HCCLM3, SMMC-7721	–	LINK-A/miR-30/MYBL2 axis	Cell proliferation, apoptosis, migration, invasion	Li et al. (2020)
Pancreatic adenocarcinoma	*In vitro*	HPAF-II, BxPC-3	–	LINK-A/ROCK1 axis	Cell proliferation, migration, invasion	Zhang et al. (2020)
Esophageal squamous cell carcinoma	*In vitro* and *in vivo*	chemoresistance cells (KYSE450/DDP, YES2/DDP) and parental cells (KYSE450, YES2)	6-week-old male BALB/c nude mice for subcutaneous xenografts, 6-week-old male NPI mice hosts for PDX tumors	FTO/LINK-A/MCM3/HIF-1a axis	Cell proliferation, cell viability, cell cycle progression, tumor metabolic reprogramming, chemoresistance	Nan et al. (2023)

HIF1α, Hypoxia-inducible factor 1-alpha; PIP3, Phosphatidylinositol (3,4,5)-trisphosphate; GPCR, G Protein-coupled receptors; PKA, Protein kinase A; TRIM71, Tripartite motif-containing 71; cAMP, cyclic adenosine monophosphate; LDH-A, Lactate dehydrogenase A; PDX, Patient-derived xenograft. The symbol “–” indicates that the information is not available or has not been reported.

Furthermore, LINK-A affects tumor growth by influencing glycolysis reprogramming (Lin et al., 2016; Wu et al., 2017; Zhao et al., 2018; Nan et al., 2023). Glycolysis reprogramming is an essential component of cancer cells’ metabolic adaptation (Faubert et al., 2020; Paul et al., 2022), and it has been reported to be involved in the activation of key molecular pathways such as the PI3K/AKT/mTOR pathway (Deng et al., 2023; Malayil et al., 2023), and the HIF-1α pathway (Nagao et al., 2019; Kierans and Taylor, 2021) in tumor progression. And LINK-A influences the glycolytic activity of cancer cells through various mechanisms, such as modulating the HIF1α signaling pathway in breast cancer (Filippov-Levy et al., 2020), interacting with the LDH-A in glioma (Wu et al., 2017), or affecting the Hexokinase-II in NSCLC(30).

Significantly, LINK-A contributes to the development of chemoresistance and immune suppression through its interaction with various key signaling pathways. For instance, LINK-A has been shown to enhance chemoresistance in cancer cells by activating the PIP3-AKT pathway (Lin et al., 2017), which promotes cell survival and inhibits apoptosis in response to chemotherapy drugs. Additionally, LINK-A can suppress immune responses by modulating the cAMP/PKA pathway (Hu et al., 2019), leading to immune evasion by cancer cells. The multifaceted role of LINK-A in regulating these pathways underscores its importance in cancer progression and therapy resistance. The following sections provide a more detailed examination of LINK-A’s multifaceted role in different cancer types.

### 2.1 Role of LINK-A in tumors of the reproductive and endocrine systems

#### 2.1.1 Breast cancer

Breast cancer is a common cancer that encompasses multiple subtypes, and treatment decisions are often guided by the presence or absence of hormone receptors (ER, PR) and HER2 [(Orrantia-Borunda et al., 2022; Turner et al., 2021)]. In contrast, triple-negative breast cancer (TNBC) is an aggressive subtype that lacks all three receptors (Lyons, 2019; Ensenyat-Mendez et al., 2021). This characteristic makes TNBC unresponsive to standard hormone or HER2-targeted therapies, making its treatment challenging. CDK inhibitors like Palbociclib have significantly advanced treatment for HR+, HER2-breast cancer by targeting CDK4 and CDK6 enzymes (Harbeck et al., 2021; Wang et al., 2024), crucial for cell proliferation (Nebenfuehr et al., 2020; Baker et al., 2022). Used alongside hormone therapy, Palbociclib has been shown to improve survival in HR+, HER2-metastatic breast cancer significantly (Serra et al., 2019; Konar et al., 2022). However, in TNBC, CDK inhibitors’ effectiveness is limited due to the absence of hormone receptors, necessitating alternative treatments like chemotherapy, targeted therapy, and immunotherapy.

In the context of breast cancer, the LINK-A lncRNA has emerged as a crucial player with significant regulatory mechanisms (Lin et al., 2016; Lin et al., 2017; Hu et al., 2019). Research conducted by Lin et al. (2016) revealed that LINK-A activates normoxic HIF1α signaling, particularly in TNBC, to be involved in tumor development. LINK-A directly participates in the signal transduction process (Lin et al., 2016). LINK-A mediates the activation of HIF1α by facilitating the recruitment and activation of BRK kinase, leading to the phosphorylation and stabilization of HIF1α, and HIF1α signaling plays a vital role in cancer cell adaptation to low oxygen levels (hypoxia) and tumor progression, contributing to glycolysis reprogramming, tumor growth and metastasis of TNBC(27). The study didn't directly examine LINK-A-HIF1α′s impact on mitochondria, but given HIF1α′s role in cellular metabolism—shifting from oxidative phosphorylation to glycolysis—it’s plausible that LINK-A could indirectly affect mitochondrial functions through HIF1α stabilization. Activation of HIF1α may alter cancer cell metabolism and mitochondrial state, suggesting indirect mitochondrial effects by the LINK-A-HIF1α axis as a potential research area.

Furthermore, Lin et al. (2017) also uncovered that LINK-A interacts with PtdIns (Wilusz et al., 2009; Chowdhary et al., 2021; Mattick et al., 2023)P3, a lipid molecule involved in cell signaling pathways, leading to the hyperactivation of the AKT pathway. This interaction causes resistance to AKT inhibitors like MK2206 and Perifosine, and enhances breast cancer cell survival and proliferation. This resistance mechanism has been demonstrated in various cell lines *in vitro* and validated *in vivo* using an athymic Nu/Nu mouse model, indicating the potent role of LINK-A in promoting drug resistance in breast cancer cells through the modulation of AKT pathway activity.

In another study by Hu et al. (2019), LINK-A was identified as an oncogenic lncRNA that downregulates cancer cell antigen presentation and intrinsic tumor suppression mechanisms. This immune evasion mechanism was investigated using various breast cancer cell lines, along with a transgenic mouse model. The study uncovered the role of LINK-A in GPCR-PKA-TRIM71 signaling and the cAMP/PKA pathway, which contribute to immuno-suppression, immunotherapy resistance, tumor growth, and metastasis.

Taken together, these findings highlight the pivotal role of LINK-A in promoting the tumorigenesis and progression of breast cancer. Studies utilizing Locked Nucleic Acids (LNA) have successfully induced the downregulation of LINK-A in tumor cells (Lin et al., 2016; Lin et al., 2017; Hu et al., 2019). Moreover, LINK-A was found to be present in fewer than 10 copies per cell in normal mammary gland epithelial cells (MCF-10A). Introducing full-length LINK-A to increase its copy number to approximately 150 per cell resulted in significant upregulation of AKT/GSK-3β phosphorylation and cell proliferation (Lin et al., 2017). While the direct effects of LINK-A knockdown in normal tissues were not explicitly explored, findings by Lin et al. (2017) suggest that lowering LINK-A levels beneath the natural baseline in cells akin to MCF-10A could reduce AKT/GSK-3β signaling activity and potentially decrease cell proliferation rates. Further investigation is required to comprehensively understand the effects of LINK-A knockdown on normal tissues, offering a promising avenue for future research. Additionally, the clinical relevance of LINK-A has been further underscored by analyses of clinical samples concerning expression and prognosis, drug interventions *in vivo* and *in vitro* following LINK-A interference, and studies on the molecular mechanisms involved in cellular signal transduction (Lin et al., 2016; Lin et al., 2017; Hu et al., 2019). Incorporating these assays into future research will not only validate LINK-A’s clinical significance in breast cancer models but also pave the way for developing targeted therapies.

#### 2.1.2 Ovarian carcinoma

The functional role of LINK-A in OC involves promoting tumor cell migration and invasion. Zhang et al. (2019b) demonstrated that LINK-A expression is significantly elevated in OC, promoting cancer cell migration and invasion, and upregulating HIF1α, suggesting its involvement in metastasis. Similarly, Ma and Xue (2018) showed that LINK-A activates the TGF-β pathway to enhance migration and invasion in OC cells. In contrast, Filippov-Levy et al. (2020) observed a different aspect of LINK-A’s function in OC. They found that LINK-A deletion resulted in increased invasiveness but reduced migration and MMP9 secretion, suggesting a potential tumor suppressor role for LINK-A in OC. However, they also noted that LINK-A deletion led to the inability of OC cells to form spheroids, indicating its importance in certain cellular processes critical for tumor progression.

These studies underscore LINK-A’s multifaceted role in OC, showcasing its potential as a unique biomarker throughout the disease’s stages. They reveal LINK-A’s dual function as both a promoter and a potential inhibitor of cancer progression, emphasizing its importance in the intricate dynamics of OC pathology and treatment strategies. Moreover, the impact of restoring LINK-A expression in downregulated samples warrants further exploration to determine if it can reverse phenotypic changes, such as enhanced invasiveness yet decreased migration, associated with LINK-A depletion. Investigating this could shed light on LINK-A’s dynamic influence on OC progression and metastasis, offering new insights into its regulatory mechanisms.

### 2.2 Role of LINK-A in tumors of the central nervous system

#### 2.2.1 Glioma

Glioma is a type of aggressive brain tumor originating in the glial cells of the central nervous system (Reni et al., 2017). The role and regulatory mechanisms of LINK-A in glioma have been investigated. Wu et al. (2017) found that LINK-A lncRNA promotes glioma cell growth and invasion via the LINK-A/LDH-A axis, which enhances cell proliferation, migration, invasion, and glycolysis. This suggests that LINK-A’s involvement in metabolic reprogramming facilitates glioma cell growth and invasive properties. In a study by Hua et al. (2019), it was demonstrated that LINK-A lncRNA participates in glioma pathogenesis through the LINK-A/Survivin axis, leading to the suppression of cell apoptosis. This interaction implies that LINK-A contributes to the survival and proliferation of glioma cells. Collectively, these findings indicate that LINK-A lncRNA plays a crucial role in promoting glioma progression by influencing metabolic pathways via the LINK-A/LDH-A axis and inhibiting cell apoptosis through the LINK-A/Survivin axis.

### 2.3 Role of LINK-A in tumors of the gastrointestinal system

#### 2.3.1 Hepatocellular carcinoma

In HCC, research conducted by Li et al. (2020) demonstrated that LINK-A is significantly upregulated in tumor samples. This overexpression correlates with advanced disease stages and a poorer prognosis in HCC patients. This suggests that LINK-A could potentially serve as a prognostic biomarker for HCC. Additionally, LINK-A acts as an oncogenic factor contributing to HCC pathogenesis. Specifically, LINK-A is involved in a complex ceRNA network. LINK-A has been shown to sequester microRNAs from the miR-30 family, effectively diminishing their regulatory effect on target mRNAs. This sequestration leads to the upregulation of MYBL2, which is known to contribute to tumor proliferation and metastasis (Musa et al., 2017; Liu et al., 2022). The dynamic interplay between LINK-A, miR-30, and MYBL2 unveils a sophisticated molecular mechanism that contributes to the aggressive characteristics of HCC. The in-depth understanding of LINK-A’s involvement in HCC progression through the ceRNA network highlights potential therapeutic targets.

#### 2.3.2 Pancreatic adenocarcinoma

In PDAC, LINK-A emerges as a crucial factor influencing vital cellular processes. Zhang et al. (2020) demonstrate that LINK-A potentially acts as an upstream regulator of ROCK1, stimulating its expression. LINK-A’s functional roles in PDAC include affecting cell proliferation, migration, and invasion; its silencing significantly curtails these cancer cell activities (Zhang et al., 2020). On the other hand, overexpression of ROCK1 can counteract the inhibitory effects of LINK-A silencing, highlighting LINK-A’s role in modulating ROCK1’s oncogenic functions. The study also suggests that the interaction between LINK-A and ROCK1 is likely indirect, influenced by disease-specific mediators, and indicative of a complex regulatory network (Zhang et al., 2020). However, the LINK-A/ROCK1 axis has not yet been identified *in vivo*, underscoring the necessity for further research to validate these findings within a physiological context. Such studies are crucial to confirm the interaction’s relevance and to understand its implications for disease progression and potential therapeutic strategies.

#### 2.3.3 Esophageal squamous cell carcinoma

For ESCC, LINK-A assumes a critical and multifaceted role in driving cancer progression and chemoresistance (Nan et al., 2023). Stabilized by the m6A demethylase FTO, LINK-A substantially impacts cell proliferation and chemoresistance in ESCC. It facilitates the interaction between the minichromosome maintenance complex component 3 (MCM3) and cyclin-dependent kinase 1 (CDK1), catalyzing enhanced phosphorylation of MCM3. This phosphorylation is key for the chromatin loading of the MCM complex, thereby advancing cell-cycle progression and cellular proliferation. Additionally, LINK-A impedes the interaction between MCM3 and hypoxia-inducible factor 1α (HIF-1α), thereby negating MCM3’s transcriptional repression of HIF-1α. This inhibition promotes increased glycolysis and chemoresistance against cisplatin in ESCC cells. These insights highlight the significance of LINK-A across various gastrointestinal cancers, underlining its potential as a therapeutic target.

### 2.4 Role of LINK-A in tumors of the respiratory system

#### 2.4.1 non-small-cell lung cancer

Lung cancer, a pivotal respiratory system tumor, is predominantly manifested as NSCLC, which constitutes about 85% of lung cancer cases (Dela Cruz et al., 2011; Zappa and Mousa, 2016). In NSCLC, LINK-A plays a crucial oncogenic role (Zhao et al., 2018; Liu et al., 2019; Maleki et al., 2021). LINK-A drives cell proliferation by modulating aerobic glycolysis via the LINK-A/Hexokinase-II axis, thus aiding in cell proliferation, aerobic glycolysis, and tumor growth (Zhao et al., 2018). Besides, the downregulation of LINK-A markedly decreased cell viability and colony-forming capabilities, as well as cell migration, while simultaneously enhancing apoptosis (Maleki et al., 2021). LINK-A is also notably upregulated in metastatic NSCLC, enhancing cell migration and invasion through Akt signaling, and is associated with poorer patient prognosis (Liu et al., 2019). The silencing of LINK-A in NSCLC cell lines like A549 and Calu-3 results in decreased cell viability, colony formation, and migration, and impacts apoptosis and cell cycle progression (Liu et al., 2019). However, understanding LINK-A’s comparative behavior and anti-tumor potency with chemotherapy in NSCLC remains unclear. Further experimental studies comparing LINK-A modulation with standard chemotherapy agents like cisplatin and docetaxel are needed. These studies will provide insights into potential synergistic or antagonistic effects, guiding the development of novel therapeutic strategies for lung cancer management.

### 2.5 Role of LINK-A in tumors of other systems

#### 2.5.1 Mantle cell lymphoma

In mantle cell lymphoma (MCL), LINK-A plays a significant role in promoting tumor progression and drug resistance (Zhang et al., 2019a; Zhang et al., 2021). LINK-A overexpression promoted cell proliferation, inhibited cell apoptosis, and upregulated survivin expression, while LINK-A knockdown had the opposite effects (Zhang et al., 2019a). Another study by Zhang et al. (2021) found that inhibition of LINK-A overcomes ibrutinib resistance in MCL by regulating the Akt/Bcl2 pathway. They found that LINK-A level was elevated in ibrutinib-resistant MCL cell lines. Functionally, LINK-A overexpression enhanced cell viability and repressed ibrutinib-induced cell apoptosis, while LINK-A knockdown decreased cell viability and accelerated ibrutinib-induced apoptosis (Zhang et al., 2021). Mechanistically, LINK-A positively regulated the activation of AKT signaling, and its inhibition overcomes ibrutinib resistance in MCL cells by affecting this pathway (Zhang et al., 2021). These findings underscore LINK-A’s function as an oncogenic factor in MCL, contributing to both tumor cell survival and resistance to chemotherapy.

#### 2.5.2 Osteosarcoma

In osteosarcoma, LINK-A plays a crucial role in tumor progression and metastasis (Zhao et al., 2019; Kong et al., 2020). Zhao et al. (2019) found that LINK-A is significantly elevated in patients with metastatic osteosarcoma, promoting cancer cell migration and invasion, and upregulating HIF1α, a factor associated with metastasis. Kong et al. (2020) further revealed that LINK-A, along with TGF-β1, is upregulated in osteosarcoma patients and positively correlated with cancer cell migration, invasion, and stemness. These studies suggest that LINK-A influences key aspects of osteosarcoma pathology, including metastasis and cell behavior, potentially through pathways involving HIF1α and TGF-β1, highlighting its importance as a potential therapeutic target and biomarker in osteosarcoma management.

## 3 Clinical implications of LINK-A in tumors

The clinical significance of LINK-A in tumors has drawn considerable attention (Lin et al., 2016; Lin et al., 2017; Filippov-Levy et al., 2018; Ma and Xue, 2018; Zhao et al., 2018; Zhang et al., 2019a; Zhang et al., 2019b; Chen et al., 2019; Hua et al., 2019; Liu et al., 2019; Zhao et al., 2019; Kong et al., 2020; Li et al., 2020; Maleki et al., 2020; Zhang et al., 2020; Ghahfarokhi et al., 2023; Nan et al., 2023), evident in its dysregulated expression profiles observed in both tumor tissues and blood samples across various malignant tumors (Table 2). Clinically, elevated levels of LINK-A are strongly associated with advanced disease stages and unfavorable prognostic outcomes, and LINK-A may also serve as a versatile diagnostic biomarker across various cancers. However, no studies directly compare LINK-A’s diagnostic accuracy with established cancer markers, such as those for breast cancer. Therefore, further research is necessary to evaluate LINK-A’s diagnostic value relative to current cancer markers. Moreover, LINK-A emerges as a potential therapeutic target, spurring research endeavors aimed at modulating or inhibiting its function to potentially mitigate or halt tumor progression.

**TABLE 2:** Expression levels of lncRNA LINK-A across diverse tumor types: correlations with clinical characteristics, prognostic implications, and diagnostic relevance.

Tumor types	Treatment received	Expression in tissues	Clinical features	Prognosis	Methods for survival analysis	Indicators for poor survival	Diagnosis	References
Breast cancer	–	Upregulated in tumor tissues	–	RFS	K-M	High expression	–	Lin et al. (2016)
	–	Upregulated in tumor tissues	–	OS	K-M	High expression	–	Lin et al. (2017)
	Yes	Upregulated in tumor tissues	–	–	–	–	–	Hu et al. (2019)
Glioma	No	Upregulated in serum of patients	–	–	–	–	AUC: 0.8543 (glioma patients vs. healthy controls)	Hua et al. (2019)
Ovarian carcinoma	Yes	–	–	OS; PFS	K-M	Low expression	-	Filippov-Levy et al. (2018)
	No	Upregulated in ovarian biopsies and plasma of patients	Distant metastasis (Plasma levels)	–	–	–	–	Ma and Xue (2018)
	No	Upregulated in ovarian biopsies and serum of metastatic ovarian carcinoma patients	Tumor metastasis	–	–	–	AUC (Tissue): 0.8898 (metastatic ovarian carcinoma patients vs. healthy controls), AUC(Serum): 0.8696 (metastatic ovarian carcinoma patients vs. healthy controls)	Zhang et al. (2019b)
	–	Upregulated in tumor tissues	Tumor stage, tumor grade	–	–	–	–	Maleki et al. (2020)
	No	Upregulated in tumor tissues	Pathological grade	–	–	–	–	Chen et al. (2019)
Non-small-cell lung cancer	–	Upregulated in tumor tissues	Tumor size, tumor grade, recurrence status	OS	K-M	High expression	–	Zhao et al. (2018)
	Yes	Upregulated in plasma of metastatic NSCLC	Tumor metastasis	OS; PFS	K-M	High expression	AUC: 0.9212 (brain-metastatic NSCLC patients vs. healthy controls), AUC: 0.9044 (bone-metastatic NSCLC vs. healthy controls), AUC: 0.9120 (liver-metastatic NSCLC vs. healthy controls)	Liu et al. (2019)
Mantle cell lymphoma	No	Upregulated in plasma of patients	–	–	–	–	AUC: 0.8338 (mantle cell lymphoma patients vs. healthy controls)	Zhang et al. (2019a)
Osteosarcoma	No	Upregulated in plasma of patients	Tumor metastasis	–	–	–	AUC: 0.9141 (metastatic osteosarcoma patients vs. healthy controls)	Zhao et al. (2019)
	No	Upregulated in plasma of patients	–	–	–	–	AUC: 0.8877 (early stage I andII patients vs. healthy controls)	Kong et al. (2020)
Hepatocellular carcinoma	No	Upregulated in tumor tissues	TNM stage, lymph node metastasis	OS	K-M, Cox multivariate analysis	High expression	–	Li et al. (2020)
Pancreatic cancer	No	Upregulated in plasma of patients	–	–	–	–	AUC: 0.8488 (pancreatic adenocarcinoma patients vs. healthy controls)	Zhang et al. (2020)
	No	–	–	–	–	–	AUC (peripheral blood MNCs): 0.57 (pancreatic cancer patients vs. healthy controls)	Ghahfarokhi et al. (2023)
Esophageal squamous cell carcinoma	Yes	Upregulated in tumor tissues	Lymph node metastasis	OS	K-M	High expression	-	Nan et al. (2023)

RFS, Recurrence-free survival; OS, overall survival; PFS, Progression-free survival; K-M, Kaplan-Meier; AUC, area under the curve; MNCs, Mononuclear cells. The symbol “–” indicates that the information is not available or has not been reported.

In the subsequent section, we delve into the clinical significance of LINK-A, concentrating on its applications in tumor diagnosis, prognosis, and treatment strategies. Our discussion covers LINK-A’s clinical values, leveraging insights from current studies and analyses of The Cancer Genome Atlas (TCGA) datasets. It is crucial to expand the scope of research and clinical trials to fully understand and utilize LINK-A’s potential in oncology.

### 3.1 LINK-A and diagnostic value

LINK-A demonstrates varied expression patterns in different tumor types, offering potential diagnostic value across diverse cancers (Zhang et al., 2019a; Zhang et al., 2019b; Hua et al., 2019; Liu et al., 2019; Zhao et al., 2019; Kong et al., 2020; Zhang et al., 2020; Ghahfarokhi et al., 2023). In glioma (Hua et al., 2019), Increased serum levels of LINK-A distinguished patients with glioma from healthy controls, showing an Area Under the Curve (AUC) of 0.8543. For metastatic ovarian carcinoma (Zhang et al., 2019b), the AUC of LINK-A expression in tumor tissue was 0.8898, and the AUC of LINK-A expression in serum was 0.8696. In metastatic non-small-cell lung cancer (NSCLC) (Liu et al., 2019), the diagnostic values of plasma LINK-A exhibited significant AUCs ranging from 0.9044 to 0.9212 in different metastatic sites. Mantle cell lymphoma (Zhang et al., 2019a) also displayed increased LINK-A levels in patient plasma, showcasing AUCs of 0.8338 for distinguishing patients from healthy controls. Notably, in early-stage osteosarcoma (Stage IandII) (Kong et al., 2020), LINK-A showed an AUC of 0.8877. For metastatic osteosarcoma (Zhao et al., 2019), increased LINK-A levels in patient plasma, showcasing AUCs of 0.9141 for distinguishing patients from healthy controls. In pancreatic adenocarcinoma (Zhang et al., 2020), elevated plasma levels were detected, yielding an AUC of 0.8488. However, in pancreatic cancer, the diagnostic value of LINK-A in peripheral blood mononuclear cells exhibited a lower AUC of 0.57(46). These findings collectively underscore LINK-A’s promising diagnostic potential across different cancers, offering unique signatures for disease detection and assessment.

**FIGURE 1:**
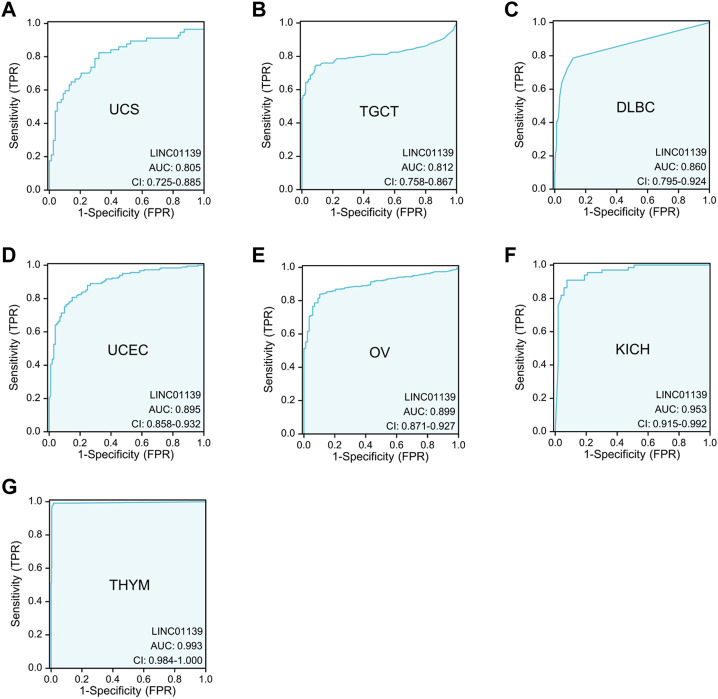
Significant diagnostic value of LINK-A in differentiating certain tumor tissues from normal tissues using TCGA_GTEx datasets. The AUC analysis demonstrates the diagnostic potential of LINK-A in distinguishing between tumorous and healthy samples across various cancer types, including UCS **(A)**, TGCT **(B)**, DLBC **(C)**, UCEC **(D)**, and OV **(E)**, with AUC values ranging from 0.8 to 0.9. Additionally, LINK-A exhibits exceptionally high diagnostic accuracy in KICH **(F)** and THYM **(G)** with AUC values exceeding 0.9.

In addition, we also performed receiver operating characteristic (ROC) curve analyses utilizing TCGA_GTEx datasets and discovered significant diagnostic value of LINK-A in distinguishing tumor tissues from normal tissues across various cancer types (Figure 1), including Uterine Carcinosarcoma (UCS), Testicular Germ Cell Tumors (TGCT), Diffuse Large B-cell Lymphoma (DLBC), Uterine Corpus Endometrial Carcinoma (UCEC), and Ovarian serous cystadenocarcinoma (OV). Notably, LINK-A expression was identified as a powerful diagnostic biomarker in kidney chromophobe (KICH) and thymoma (THYM), with AUC values surpassing 0.9. These findings underscore the potential of LINK-A as a promising diagnostic predictor in a broad spectrum of malignancies.

Despite these promising findings, it is crucial to note that there are no comparative studies of LINK-A’s AUC values against those of established molecular biomarkers yet. This underscores the necessity for future research aimed at conducting comparative analyses between LINK-A and established biomarkers to better understand LINK-A’s diagnostic capacity and potential advantages over existing markers.

### 3.2 LINK-A and prognostic significance

The lncRNA LINK-A exhibits significant prognostic implications across various cancers (Lin et al., 2016; Lin et al., 2017; Filippov-Levy et al., 2018; Ma and Xue, 2018; Zhao et al., 2018; Zhang et al., 2019b; Chen et al., 2019; Liu et al., 2019; Li et al., 2020; Maleki et al., 2020; Nan et al., 2023). In breast cancer (Lin et al., 2016; Lin et al., 2017), elevated LINK-A expression in tumor tissues correlated with poorer recurrence-free survival and shorter overall survival. Conversely, in high-grade serous carcinoma (Filippov-Levy et al., 2018), low LINK-A expression in tumor tissues was associated with reduced overall survival and inferior progression-free survival. Multiple studies (Ma and Xue, 2018; Zhang et al., 2019b; Chen et al., 2019; Maleki et al., 2020) highlighted the upregulation of LINK-A in OC patients’ biopsies and plasma, with its heightened expression significantly correlating negatively with tumor grade, metastasis, and stage. Similarly, in NSCLC(30, 33), increased LINK-A expression was observed not only in solid tumor tissues but also in the plasma of patients with metastasis. This elevated expression level is closely associated with advanced clinical features, including larger tumor size, higher tumor grade, and tumor metastasis (Zhao et al., 2018; Liu et al., 2019). Additionally, patients with high LINK-A expression exhibited a poorer prognosis (Zhao et al., 2018; Liu et al., 2019). In osteosarcoma, elevated LINK-A expression observed in patients’ plasma was positively related to tumor metastasis (Zhao et al., 2019). Furthermore, in HCC(44) and ESCC(47), elevated LINK-A levels in tumor tissues were indicative of lymph node metastasis and reduced overall survival.

**FIGURE 2:**
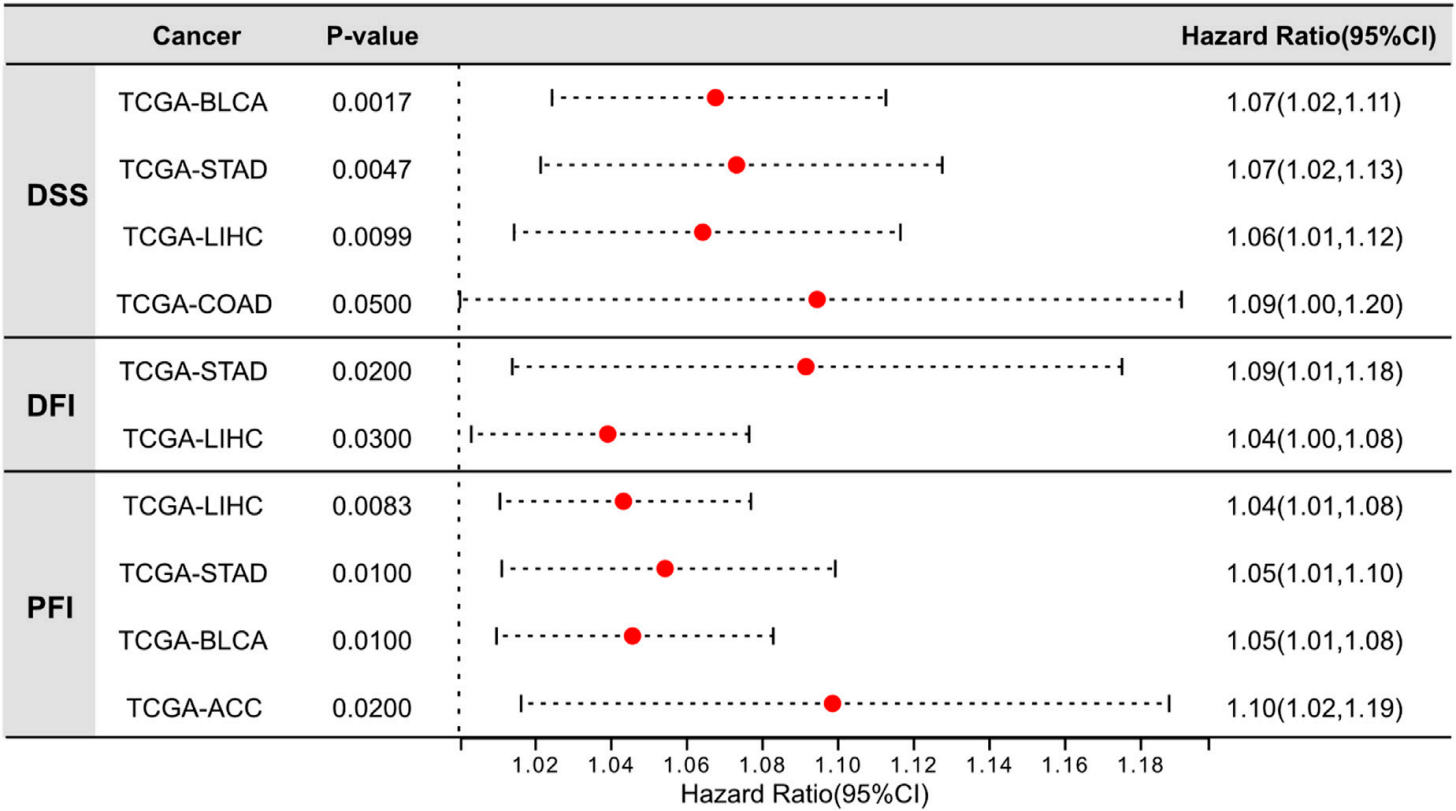
Significant prognostic value of LINK-A expression in tumor tissues of specific cancer types. Analysis of the relationship between LINK-A overexpression and Disease-Specific Survival, Disease-Free Interval, and Progression-Free Interval across various malignancies. Data sourced from the UCSC Xena Browser (https://xenabrowser.net/).

Moreover, we further assessed the association between LINK-A expression levels and disease-specific survival (DSS), disease-free interval (DFI), and progression-free interval (PFI) across a range of malignancies (Figure 2), utilizing TCGA datasets. Our findings revealed a significant association between LINK-A expression levels and DSS in bladder urothelial carcinoma (BLCA) and stomach adenocarcinoma (STAD), liver hepatocellular carcinoma (LIHC) and colorectal adenocarcinoma (COAD). Furthermore, notable associations were found between high LINK-A expression and reduced DFI in STAD and LIHC, as well as poorer PFI in LIHC, STAD, BLCA, and adrenocortical carcinoma (ACC), highlighting LINK-A’s potential as a prognostic indicator in these malignancies.

### 3.3 LINK-A and therapeutic targets

LINK-A, a multifaceted lncRNA, has emerged as a promising therapeutic target in cancer due to its significant role in tumorigenesis. The application of RNA interference (RNAi) technologies, such as Small Interfering RNA (siRNA) and Short Hairpin RNA (shRNA), for silencing or knocking down LINK-A, has demonstrated potential in impeding cancer cell activities and curtailing tumor growth and metastasis.

The versatility of LINK-A as a therapeutic target is evident in its varied roles in different cancers, ranging from promoting tumor progression to modulating cellular behaviors such as cell cycle, proliferation, migration, and invasion. Furthermore, LINK-A’s interaction with several key signaling pathways, including HIF1α, PIP3-AKT, cAMP/PKA, TGF-β, and ERK, underscores its significant impact on cancer biology, highlighting LINK-A as a potential target for further exploration.

Notably, LINK-A has been implicated in the reprogramming of glycolysis, targeting LINK-A has the potential to disrupt glycolysis, potentially slowing down cancer progression by depriving cells of the energy they need. Specifically, in TNBC(27), LINK-A activates HIF1α signaling, thereby enhancing glycolysis and providing a survival advantage to cancer cells. In glioma (Wu et al., 2017), LINK-A regulates LDH-A, and its enforced expression leads to increased glycolysis, characterized by heightened glucose uptake and lactate production. This indicates the involvement of the LINK-A/LDH-A axis in the glycolysis of glioma cells. Further, in NSCLC(30) and ESCC(47), LINK-A is known to promote oncogenic processes such as cell proliferation and metastasis by modulating glycolysis. Additionally, a KEGG pathway analysis conducted with the lnCAR program identified LINK-A as being associated with both metabolic pathways and pathways in cancer (Li et al., 2020), highlighting the broad impact of LINK-A on cancer metabolism and progression.

In the context of drug resistance, LINK-A’s involvement is noteworthy. It has been implicated in promoting resistance to targeted therapies. For example, in breast cancer (Lin et al., 2017), it has been discovered that LINK-A acts as a crucial protein scaffold, engaging directly with both the AKT pleckstrin homology domain and PIP3 at the single nucleotide level. This interaction leads to the enzymatic activation that plays a pivotal role in both the development of tumors and resistance to treatments targeting the AKT pathway. Intriguingly, experiments have shown that removing the specific PIP3 binding domain within LINK-A can make breast cancer cells significantly more responsive to AKT inhibitor treatments. Similarly, in MCL(42) and ESCC(47), LINK-A overexpression has been associated with increased resistance to chemotherapy.

Furthermore, LINK-A has been linked to immune suppression and resistance to immunotherapy in TNBC(36). LINK-A’s activation of HIF1α signaling may contribute to an immunosuppressive tumor microenvironment, challenging the immune system’s ability to mount an effective antitumor response. Targeting LINK-A in these scenarios may sensitize cancer cells to therapeutic agents, potentially overcoming drug resistance and improving treatment outcomes.

## 4 Future perspectives

**FIGURE 3:**
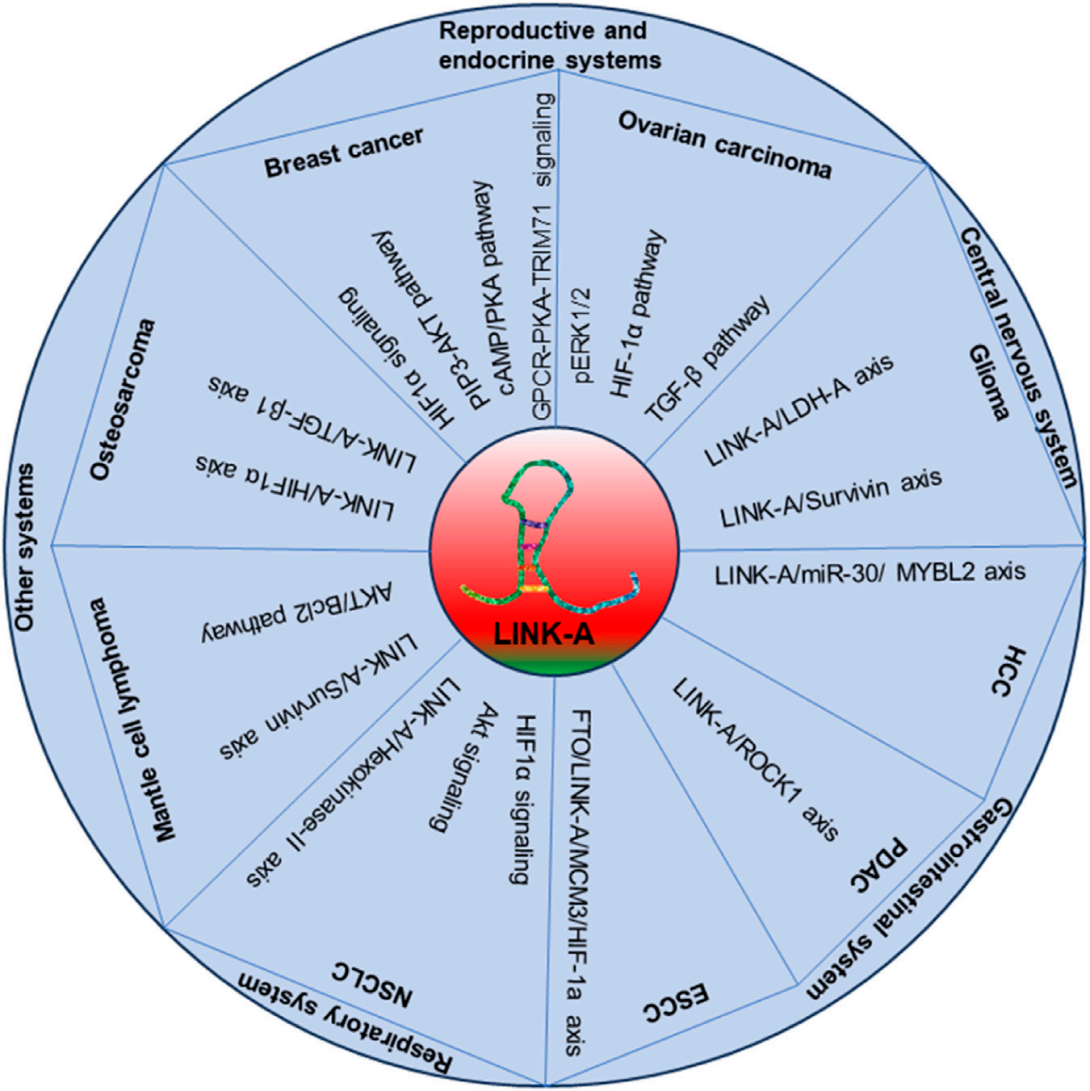
The illustrative model detailing the role of LINK-A in cancer development and progression.

The multifaceted lncRNA LINK-A has emerged as a promising and pivotal player in multiple cancers across different systems. A comprehensive understanding of LINK-A’s molecular mechanisms in various cancer types is essential, as shown in Figure 3. Its diverse roles in tumorigenesis, tumor progression, and therapeutic resistance have positioned it as a significant molecule in the landscape of cancer biology. LINK-A impacts various aspects of cancer, including cell behavior regulation, metabolic reprogramming, and immune modulation.

However, the upstream and downstream regulatory mechanisms of LINK-A, particularly in cancers where its functions remain enigmatic, require further investigation. Exploring LINK-A’s interactions with more signaling pathways, microRNAs, and proteins is crucial. In-depth mechanistic studies, both *in vitro* and *in vivo*, can provide more insights into LINK-A’s role in different cancers. Uncovering these regulatory networks may unveil novel therapeutic targets and diagnostic markers. These investigations should be conducted rigorously and validated in clinical contexts to translate findings into practical applications.

**FIGURE 4:**
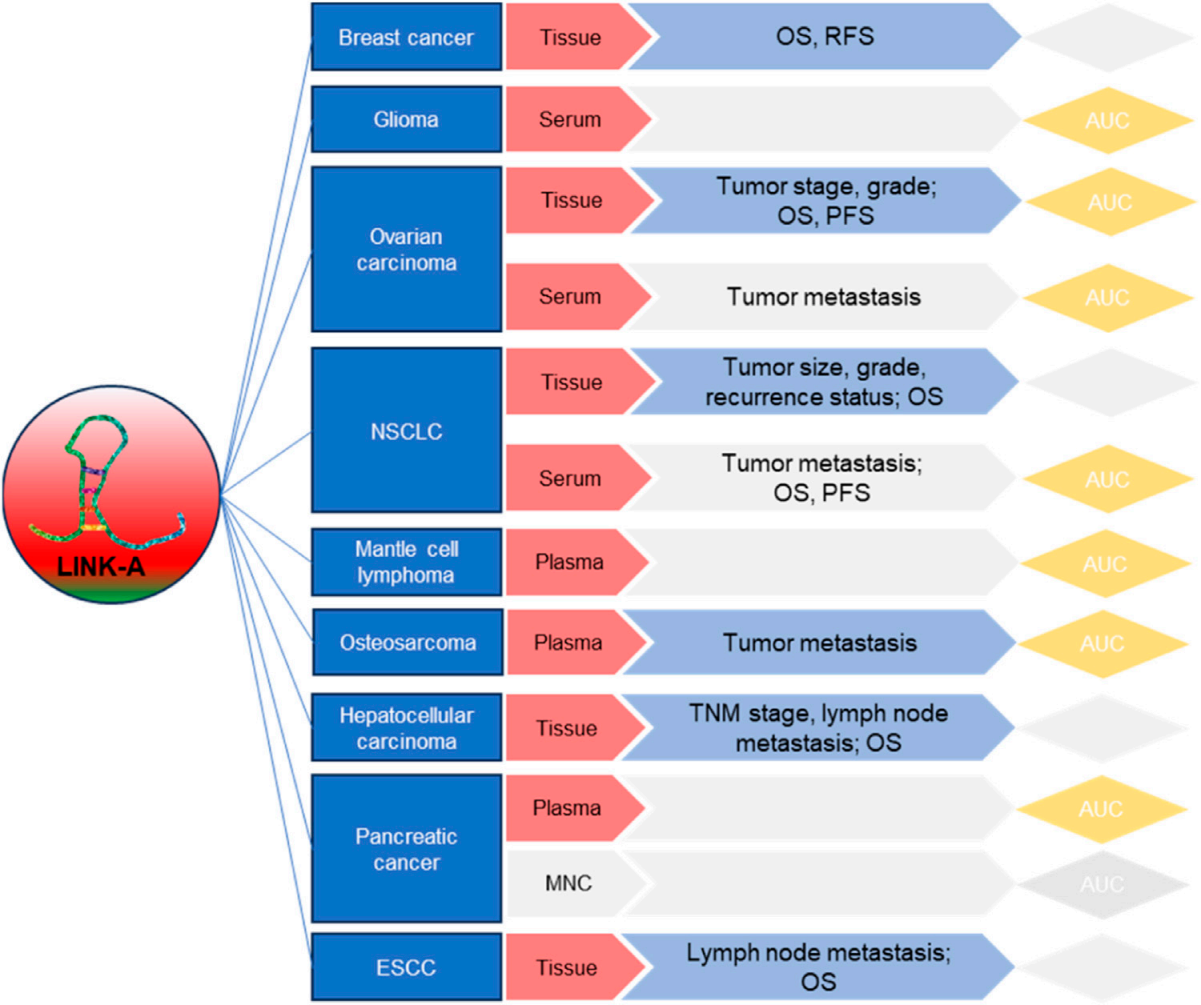
Summary of LINK-A’s clinical relevance, prognostic, and diagnostic applications in cancers.

Moreover, the development of targeted therapies aimed at LINK-A holds immense potential for improving cancer treatment outcomes. By addressing LINK-A’s roles in drug resistance, glycolysis, and immunosuppression, novel therapeutic strategies can sensitize cancer cells to existing treatments and enhance the effectiveness of immunotherapies. Clinical trials and preclinical studies should be conducted to evaluate the safety and efficacy of these interventions. Additionally, numerous studies have highlighted the diagnostic and prognostic potential of LINK-A (Figure 4). Yet, the utility of LINK-A in these aspects demands thorough validation in more clinical cohorts. Its capability to predict disease progression, response to therapy, and patient outcomes holds promise for transforming personalized cancer care. Incorporating LINK-A expression profiles into clinical practice could significantly improve patient management strategies.

In our review, we have highlighted the clinical significance and the molecular pathways regulated by LINK-A in cancer. Recognizing the potential of LINK-A as a therapeutic target, it is crucial to acknowledge the current research gap in direct comparative studies of its downregulation, particularly through antisense oligonucleotides (ASOs) or other molecular interventions, compared with established cancer treatments. The absence of such studies may reflect the nascent stage of LINK-A targeted therapy research and the complexity involved in conducting these comparative analyses. Future research directions should aim to fill this gap by developing rigorous experimental designs to evaluate the efficacy of LINK-A inhibition compared to gold-standard cancer therapies. While our review outlines the theoretical rationale and potential of targeting LINK-A based on its involvement in critical oncogenic pathways, empirical evidence from comparative studies would significantly contribute to validating LINK-A as a viable therapeutic target. The exploration of LINK-A’s therapeutic potential offers a promising avenue for novel cancer treatment strategies, underscoring the importance of continued investigation in this field.

## 5 Conclusion

In summary, LINK-A has emerged as a novel lncRNA with a diverse role in the pathogenesis and progression of cancer. Future research delving into its intricate molecular mechanisms and potential clinical applications is pivotal. Such exploration promises to enhance our understanding of LINK-A’s role in oncology, potentially revolutionizing approaches to cancer diagnosis, prognosis, and therapy. It positions LINK-A as a key player in innovative cancer treatment strategies.
